# *In Vivo* Volume Dynamics of Dendritic Spines in the Neocortex of Wild-Type and *Fmr1* KO Mice

**DOI:** 10.1523/ENEURO.0282-18.2018

**Published:** 2018-11-08

**Authors:** Kazuhiko Ishii, Akira Nagaoka, Yutaro Kishida, Hitoshi Okazaki, Sho Yagishita, Hasan Ucar, Noriko Takahashi, Nobuhito Saito, Haruo Kasai

**Affiliations:** 1 Laboratory of Structural Physiology, Center for Disease Biology and Integrative Medicine, Graduate School of Medicine, The University of Tokyo, Bunkyo-ku, Tokyo 113-0033, Japan; 2 Department of Neurosurgery, Graduate School of Medicine, The University of Tokyo, Bunkyo-ku Tokyo 113-0033, Japan; 3 International Research Center for Neurointelligence, The University of Tokyo Institutes for Advanced Study, The University of Tokyo, Bunkyo-ku, Tokyo 113-0033, Japan; 4 Department of Physiology, Kitasato University School of Medicine, Sagamihara 252-0374, Japan

**Keywords:** ASD, autism, dendritic spine, fragile X mental retardation, synaptic plasticity, two-photon microscopy

## Abstract

Excitatory synapses are often formed at small protrusions of dendrite, called dendritic spines, in most projection neurons, and the spine-head volumes show strong correlations with synaptic connectivity. We examined the dynamics of spine volume in the adult mouse visual cortex using time-lapse *in vivo* two-photon imaging with a resonant Galvano scanner. Contrary to expectations, we found that the spines in the adult neocortex showed fluctuations to a similar degree as that observed in young hippocampal preparations, but there were systematic differences in how the dynamics were dependent on spine volumes, thus allowing for fewer fluctuations in small spines, which could account for the relatively low turnover rates of neocortical spines *in vivo*. We found that spine volumes fluctuated to a greater extent in a mouse model (*Fmr1* knockout) of fragile X mental retardation than in wild-type mice, and the spine turnover rates were also higher in *Fmr1* knock-out mice. Such features of spine dynamics in *Fmr1* knock-out mice could be represented by a single slope factor in our model. Our data and model indicate a small but significant change in the average spine volume and more eminent differences in the statistical distribution in *Fmr1* knock-out mice even in adulthood, which reflects the abnormal *in vivo* dynamics of spine volumes.

## Visual Abstract

**Figure:**
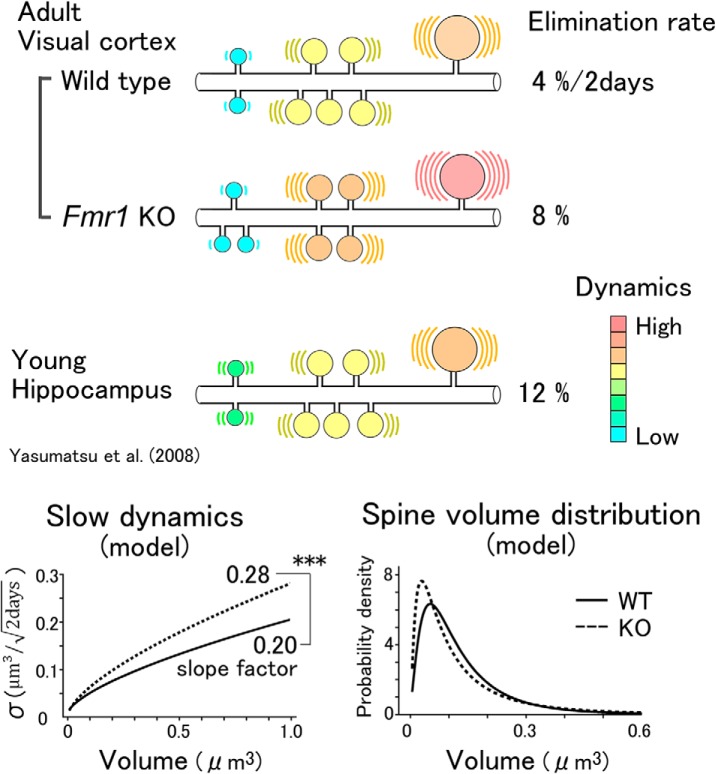

## Significance Statement

Excitatory synapses are often formed at dendritic spines in projection neurons, and the spine volumes show strong correlations with synaptic connectivity. We studied the dynamics of spine volumes *in vivo* using a resonant scanner two-photon microscope and found that spine dynamics showed a distinctive dependence on spine volume in the adult neocortex, allowing for fewer fluctuations in these small spines than in the spines of young hippocampi. Moreover, the dynamics of spine volumes explained the greater turnover rate of spines and the smaller spine volume observed in a mouse model (*Fmr1* knockout) of fragile X syndrome mental retardation than in wild-type mice. Our results indicate that the dynamics of spine volumes are abnormal in *Fmr1* knock-out mice, even in adulthood.

## Introduction

Excitatory glutamatergic synapses are formed at small protrusions of dendrites, called dendritic spines. As dendritic spines form synapses, their generation, enlargement, shrinkage, and elimination underlie the formation and maintenance of neuronal networks, and their head sizes strongly correlate with synaptic efficacy (Matsuzaki et al., 2001, 2004; Noguchi et al., 2005; Béïque et al., 2006; Bhatt et al., 2009; Holtmaat and Svoboda, 2009; Kasai et al., 2010; Herring and Nicoll, 2016; Yasuda, 2017; Moyer and Zuo, 2018). The morphology and/or dynamics of dendritic spines have been found to be impaired in some psychiatric diseases (Fiala et al., 2002; Penzes et al., 2011), particularly autism spectrum disorder (ASD; Purpura, 1974). Fragile X syndrome is one of the most prevalent monogenic forms of ASD and is caused by the expansion of CGG repeats in the *Fmr1* gene, which encodes the Fragile X Mental Retardation Protein (Consortium-D-BFX, 1994). In the fragile X mental retardation, dendritic spines are long, thin, and tortuous (Rudelli et al., 1985), suggesting that they are immature (He and Portera-Cailliau, 2013).

*Fmr1* gene knock-out (KO) mice show abnormalities in learning and neuronal plasticity (Consortium-D-BFX, 1994; Comery et al., 1997; Cruz-Martín et al., 2010; He and Portera-Cailliau, 2013; Padmashri et al., 2013; Sidorov et al., 2013). These mice also show increases in the turnover rate of spine, which reflects the generation and elimination of spines (Pan et al., 2010; Padmashri et al., 2013; Nagaoka et al., 2016). Dendritic spines are found to be smaller in juvenile *Fmr1* KO mice (Cruz-Martín et al., 2010; Pan et al., 2010; Swanger et al., 2011; He and Portera-Cailliau, 2013; Lauterborn et al., 2015), but the differences are not prominent in adult mice (Nimchinsky et al., 2001; Wijetunge et al., 2014). No previous study, however, has measured the changes in spine volumes and examined their correlations with the spine volume distributions in the mutant, because the quantification of volume dynamics was technically more demanding than that of spine turnover. Some groups have succeeded in the measurement of spine volume changes in wild-type (WT) mice (Yasumatsu et al., 2008; Loewenstein et al., 2011; Statman et al., 2014) and revealed that such dynamics provide a good basis for the volume distribution and turnover of spines using mathematical models with stochastic processes (random walks).

Here, we investigated the differences in the dynamics of dendritic spines in WT and *Fmr1* KO adult mice *in vivo*. We used a two-photon microscope equipped with a resonant scanner to overcome the limitation of the inherent pulsations in brains *in vivo*. We applied the same model that was developed for the rat hippocampal slice preparations *in vitro* (Yasumatsu et al., 2008). We found that the spine fluctuations depended on the volume of spines in the adult neocortex, which differs from what is observed in the young hippocampus, which could account for the relative stability of the spines in the neocortex. It could also explain the difficulty in detecting the difference in the average spine volumes between adult WT and *Fmr1* KO mice in previous reports (Nimchinsky et al., 2001; Wijetunge et al., 2014). Our study clarified the critical impairments in spine dynamics and other spine parameters in *Fmr1* KO mice even in adulthood.

## Materials and Methods

### Animals

A colony of homozygous transgenic mice expressing a green fluorescent protein (GFP) under the control of the Thy1 promoter (*Thy1*-GFP M-line mice) was generated for the present study. B6.129P2-*Fmr1^tm1Cgr^*/J (The Jackson Laboratory) KO female mice (*Fmr1^+^* ^/−^) were crossed with males homozygous for *Thy1*-GFP to generate GFP-*Fmr1*−*/y* mice. Adult male mice >2 months of age were anesthetized with isoflurane (4.5% for induction; 0.8–1.5% for maintenance) during the operation. The level of anesthesia was assessed by monitoring the tail-pinch reflex. The administration of 20% mannitol (20 μl/g body weight) was performed intraperitoneally, while ketoprofen (2 μl/g body weight) was administered subcutaneously. A head plate with a 5-mm-diameter hole was fixed to the skull using dental cement (Fuji Lute BC, GC). A small craniotomy (diameter, 2.7 mm) was performed over the left visual cortex based on stereotaxic coordinates (posterior, 3.0 mm from the bregma; lateral, 2.5 mm from the bregma; Paxinos, 2001), which were confirmed by intrinsic signal imaging. The craniotomy was performed using a trephine drill (catalog #224RF-027, Meisinger) fixed to a stereotaxic instrument. A circular glass (diameter, 2.7 mm; Matsunami) was fixed to the cranial window using the dental cement (Fuji Lute BC, GC) and a dental acrylic device (ADFA, Shofu) to the cranial window.

**Figure 1.**
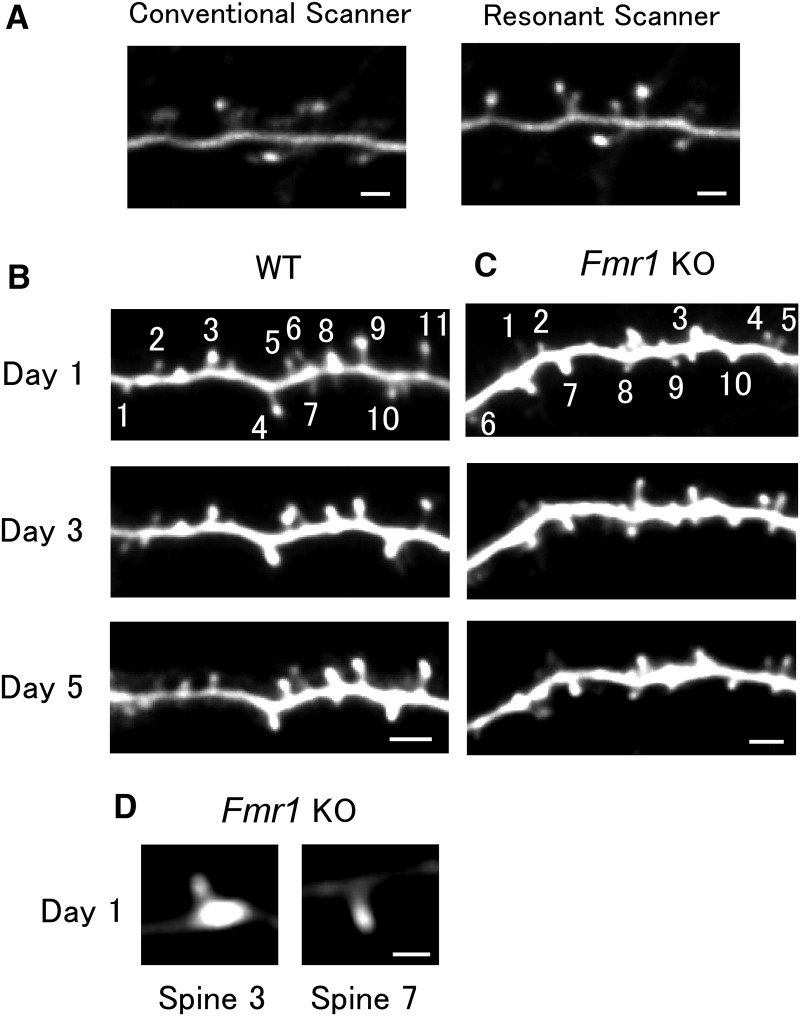
Apical dendritic spines of M-line mice in the mouse visual cortex imaged at an interval of 2 d. ***A***, Sample images of a mouse with considerable pulsation artifacts obtained with conventional and resonant Galvano scanners. The resonant scanner was relatively robust in response to motion artifacts. Scale bar, 2 μm. ***B***, ***C***, Examples of a time-lapse series of dendrite images from WT (***B***) and KO (***C***) mice. Spine numbers correspond to the respective traces in the volume–time courses in Figure 2, *A* and *B*. Scale bar, 3 μm. ***D***, Spines 3 and 7 in ***C*** are displayed at a different brightness level from that used in ***C***. Scale bar, 1 μm.

**Figure 2.**
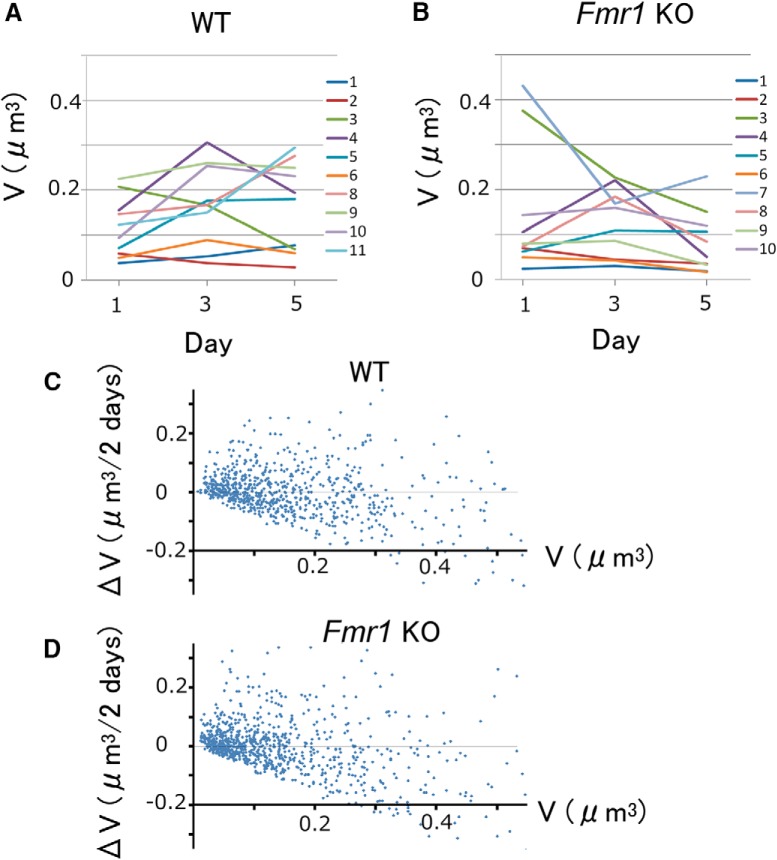
Changes in spine volumes in the mouse visual cortex per 2 d. ***A***, ***B***, Time course of spine volume changes for the spines shown in Figure 1, *B* and *C*. Trace numbers correspond to the spines numbered in Figure 1, *B* and *C*. ***C***, ***D***, Spine-head volume changes (Δ*V*) per 2 d in WT (***C***) and *Fmr1* KO (***D***) mice. Data were obtained from 754 spines from 15 dendrites in five WT mice and from 878 spines from 20 dendrites in five KO mice.

Imaging experiments were performed at least 1 d after the surgery and repeated every other day for 3–23 d (2–12 imaging sessions) as long as the conditions of the animal, cranial window, brain, and dendrites remained stable. Data were pooled and analyzed together. The most frequent contiguous imaging duration was 5 d. Examples of images obtained in this duration are shown in Figure 1, *B*and *C*, with the corresponding spine volume time course shown in Figure 2, *A* and *B*. After imaging, mice were returned to their respective cages. Five KO mice and five WT (littermates) mice were examined, and one to five dendrites were imaged for each mouse. Images were obtained from five other mice in a similar manner at intervals of 10 min. All animal procedures were performed in accordance with the regulations of The University of Tokyo Animal Care Committee.

### Two-photon imaging

Two-photon imaging was performed using an upright microscope (models BX61WI and FV1000-MP, Olympus) equipped with a resonant scanner and a GaAsP detector with a 25× (1.05 numerical aperture; catalog #XLPLN25XWMP2, Olympus) water-immersion lens. A Ti-sapphire laser (Mai-Tai-DS-HP, SpectraPhysics) was tuned to 950 nm wavelength for the excitation of the GFP. The average excitation power was maintained at <40 mW under the objective. The power was selected to avoid saturating the fluorescence of dendrites. Mice were anesthetized with a 0.8–1.5% isoflurane–oxygen mixture (Univentor 400 anesthesia unit, Univentor), and the body temperature was maintained at 37°C using a heating pad. During the image acquisition, mice were restrained using a head plate. Image stacks were obtained from the apical dendritic tufts of layer V pyramidal in the layer I (20–70 μm from the pial surface), and, typically, 20–40 frames (512 × 512 pixels; 0.124 μm/pixel; 17 ms/frame) per stack were obtained at an interval of 0.4 μm along the *z*-axis. Each dendrite was traced to the soma to confirm its layer of origin.

### Measurements of spine dynamics

Z-stack images were aligned using StackReg in ImageJ (RRID:SCR_003070), and summed over the *z*-direction. Only spines that were well separated from the dendrite were included in the analysis. A region of interest (ROI) surrounding the spine head was chosen manually, and another ROI was placed at the same distance from the dendrite where there were no spines, to obtain the background fluorescence level, which was then subtracted from the fluorescence of the ROI over the spine. The total fluorescence (*F*) of a spine head was considered to be related to spine-head volume (*V*), assuming that GFP homogeneously filled the volume of the spine, as previously described (Matsuzaki et al., 2004, Supplementary Methods). To this end, the largest and most spherical spine on the dendrite was selected from the data obtained in an early imaging session of each dendrite, and the one-dimensional fluorescence profile across the center of the spine head was measured. This was fitted by the following equation for the fluorescence intensity profile of spherical objects of radius *R* (Eqn. 1):

(1) $$F\left(r,R\right)=\frac{A}{{\left(2\pi \right)}^{3/2}{\sigma_{x}}^{2}\sigma_{z}}\overset{}{\underset{x^{2}+y^{2}+z^{2}<R^{2}}{\int \int \int }}dxdydz\int dz^{'}\,\;e^{-[\frac{{\left(x-r\right)}^{2}}{2\sigma_{x}^{2}}+\frac{y^{2}}{2\sigma_{x}^{2}}+\frac{{\left(z-z'\right)}^{2}}{2\sigma_{z}^{2}}]},$$

where *A* represents the intensity at the center (*r* = 0) of the sphere. Point spread functions were estimated to be 0.56 μm full-width at half-maximum (σ*_x_* = 0.24 μm) laterally and 2.1 μm (σ*_z_* = 0.91 μm) axially based on the images of the smallest structures such as filopodia in our own experiments *in vivo*. The volume of the spine was thus calculated as *V* = 4/3 π*R* ^3^, and its ratio (*V*/*F*) to the total fluorescence was used for calculating spine volume from its total fluorescence in the same field for all subsequent imaging sessions. This conversion coefficient could be used to estimate spine volumes irrespective of their fine structures. To correct for day-to-day variation in the expression of GFP, total fluorescence of the dendritic region was recorded for each sample on each day. Estimates of spine-head volume were corrected for this variation, which was usually <10% per day (range, 0.3–25%; Yasumatsu et al., 2008).

Filopodia (protrusions with head diameter/neck diameter ratio, <1.2; length/neck diameter ratio, >3) were excluded from the analysis. For the measurement of elimination rates, original images were carefully examined to confirm the elimination to avoid misleading interpretations of spine elimination for spines moving behind dendrites or overlapping with a neighbor. Images not bright and clear enough to enable unambiguous distinction were excluded from the measurement of elimination rates.

### Mean and SD of changes in spine-head volume

The mean change in spine-head volume [μ(*V*)] and the SD of this change [σ(*V*)] were calculated per 2 d using the following equations (Eqn. 2, 3):

(2) $$\sigma^{2}\left(R_{j}\right)=\sum_{i,k|V_{k}(i)\in R_{j}}{\left(V_{k}\left(i+1\right)-V_{k}\left(i\right)-\mu (R_{j})\right)}^{2}/N_{j}$$

(3) $$\mu \left(R_{j}\right)=\sum_{i,k|V_{k}(i)\in R_{j}}{\left(V_{k}\left(i+1\right)-V_{k}\left(i\right)\right)}^{}/N_{j},$$

where each value of μ(*V*) and σ(*V*) was obtained for individual imaging sessions (*i* = 1,2,…) and where spines (*k* = 1,2,…) had volumes of *V_k_*(*i*) falling within each of the volume bins *R_j_* (*j* = 1,2,…,24). *N_j_* is the total number of spines satisfying *V_k_*(*i*) ϵ *R_j_*.

**Figure 3.**
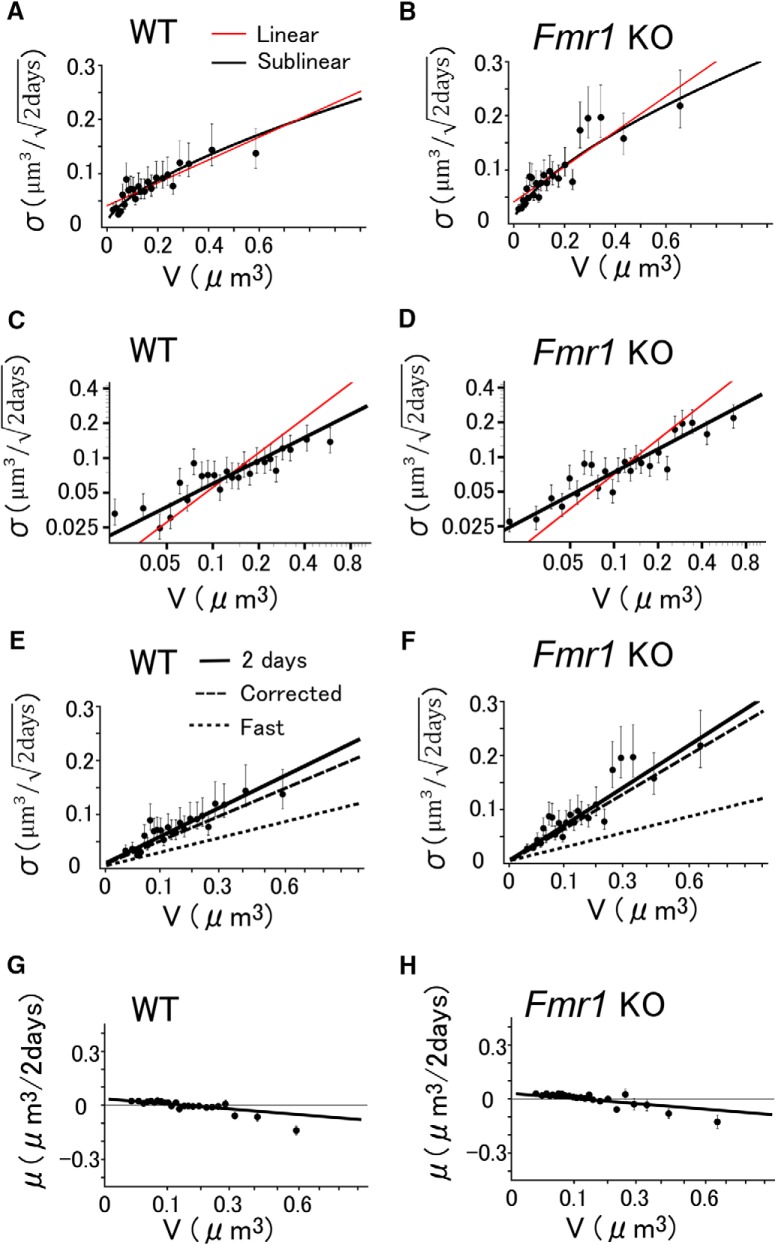
Slow dynamics of spine-head volume measured per 2 d. ***A***, ***B***, The dynamics of spine-head volumes in WT (***A***) and KO (***B***) mice plotted against *V*. Each plotted point represents the SD of spine-head volume changes in 32 pooled spines of similar volume. Error bars represent 95% confidence intervals of the estimated SD. The red lines represent the least squares fits. The red line for WT mice (***A***) is represented by 0.21*V* ^2/3^ + 0.041, and for KO mice (***B***) it is represented by 0.32*V* ^2/3^ + 0.041. Note that the values of σ were below the red lines for small spines. ***C***, ***D***, Double logarithmic plots of the dynamics of spine-head volumes shown in ***A*** and ***B***. Each plotted point represents the SD of spine-head volume changes in 32 pooled spines of similar volume. Error bars represent 95% confidence intervals of the estimated SD. The black lines with a slope of two-thirds fit well with the data. The red lines are reference lines with a slope of 1. ***E***, ***F***, Slow spine-head volume changes in WT (***E***) and *Fmr1* KO (***F***) mice plotted against *V* ^2/3^ (note that the numbers and scales on the *x*-axis represent *V*). Each plotted point represents the SD of spine-head volume changes in 32 pooled spines of similar volume. The solid line is a fit to the data, and can be decomposed into a slow component (dashed lines; Eq. 6, confidence interval of the slope 0.18–0.22) and a fast component, obtained using measurements at 10 min intervals (dotted lines; Eq. 4) for ***E***, and Equation 7 (95% confidence interval of the slope 0.24–0.32) for ***F***. The slope of the fitted line for KO mice is significantly larger than that for WT mice (generalized linear model, *t* statistic = −3.67, *p* = 0.0006^a^). ***G***, ***H***, The drift of the spine-head volume changes in WT (***G***) and *Fmr1* KO (***H***) mice plotted against the initial *V* value. The abscissa is linear in *V* ^2/3^, while the numbers and scales represent *V* as in ***E*** and ***F***. Each plotted point represents the mean of spine-head volume changes in 32 pooled spines of similar volume. Error bars represent the SEM. The lines are the same for the pooled data in WT and KO mice (Eq. 8).

**Figure 4.**
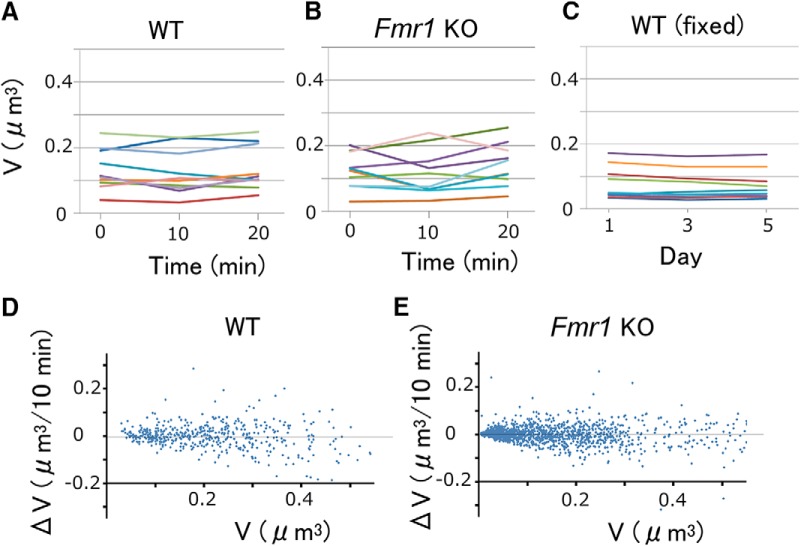
Fast volume changes in apical dendritic spines in the mouse visual cortex. ***A***, ***B***, Spine-head volume changes in WT (***A***) and KO (***B***) mice measured at an interval of 10 min. ***C***, Spine-head volume changes in a fixed sample imaged at an interval of 2 d. ***D***, ***E***, Head volume changes (Δ*V*) per 10 min in WT (***D***) and *Fmr1* KO (***E***) mice. Data were obtained from 475 spines from two dendrites in two WT mice, and from 1433 spines from four dendrites in three KO mice.

**Figure 5.**
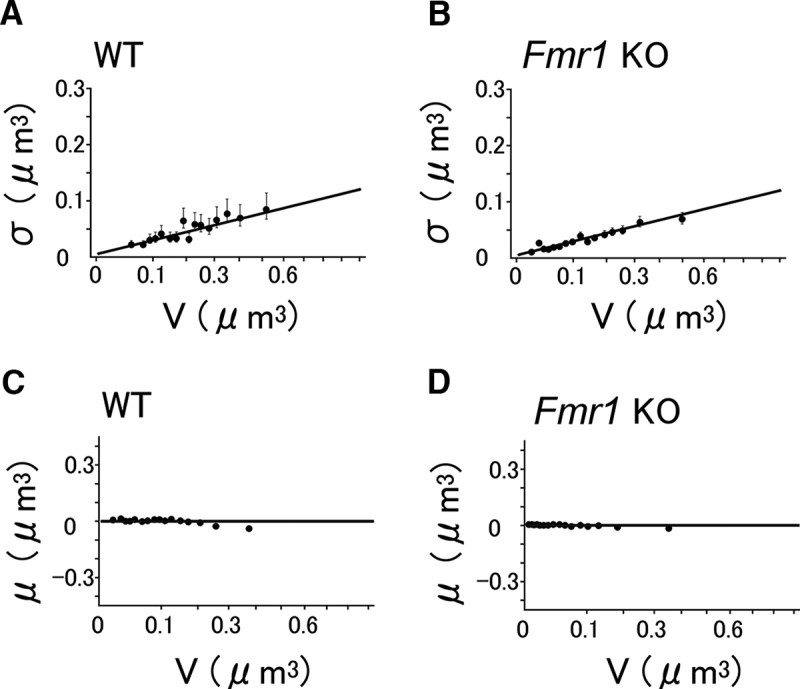
Fast dynamics of spine-head volume. ***A***, ***B***, The fast component of the dynamics of spine-head volume in WT (***A***) and KO (***B***) mice plotted against *V* ^2/3^. Each plotted point represents the SD of spine-head volume changes in 30 pooled spines of similar volume. Error bars represent 95% confidence intervals of the estimated SD. The line represents the least squares fit of the pooled data for WT and KO (Eq. 4). ***C***, ***D***, Fast component of the drift of spine-head volume in WT (***C***) and KO (***D***) mice plotted against *V* ^2/3^. Each point represents the mean of spine volume changes in 30 spines of similar volume. Error bars represent SEM values. Because the drifts were very small, they were fitted by zero (line).

The values of σ defined by Equation 2 included the fast fluctuations by the rapid actin dynamics and measurement errors, which should be treated differently from the long-term behaviors of spines. We thus obtained such rapid fluctuations, $\sigma_{fast}$, by imaging spine volumes every 10 min for 1 h (see Fig. 4). The mean values (μ) were zero, consistent with the nature of the fast fluctuations, and $\sigma_{fast}$ could be fitted by the following equation (Fig. 3*E*,*F*, dotted lines; and see Fig. 5, dotted lines) (Eqn. 4):

(4) $$\sigma_{fast}=0.115V^{2/3}+0.0051.$$

For simplicity, we expressed the values as the SD of the sample in Figure 5, as the means were zero, and the variation did not depend on the sampling interval by definition (Matsuzaki et al., 2004; Yasumatsu et al., 2008). The total dynamics were the sum of these fast dynamics and the slow dynamics ($\sigma_{slow}$) that determined the long-term behaviors of the spine, as follows (Eqn. 5):

(5) $$\sigma^{2}=\sigma_{fast}^{2}+\sigma_{slow}^{2}.$$

The dynamics of σ were plotted against *V* in a double logarithmic plot, as seen in Figure 3, *C*and *D*. We noted that the slope was less than one (red line), and nearly two-thirds (black line). Therefore, in the following graphs, μ and σ were plotted against *V* ^2/3^. A regression line for the slow dynamics ($\sigma_{slow}$) calculated using Equation 5, and the experimentally obtained σ and $\sigma_{fast}$ was plotted against *V* ^2/3^, as seen in Figure 3*E* (dashed line), and was expressed as follows (Eqn. 6):

(6) $$\sigma_{slow}\left(V\right)=0.198\left(V^{2/3}-0.06\right)+0.020=0.198V^{2/3}+0.0081.$$

For *Fmr1* KO mice, it was as follows (Eqn. 7):

(7) $$\sigma_{slow}\left(V\right)=0.278\left(V^{2/3}-0.06\right)+0.020=0.278V^{2/3}+0.0033.$$

The slow drift μ(per day) was obtained in a similar manner in both WT and *Fmr1* KO mice, as shown below (Eqn. 8):

(8) $$\mu \left(V\right)=-0.12V^{2/3}+0.029.$$

To reassure that the operation itself did not affect the results, the analysis was performed excluding imaging data from day 1 postsurgery. The slope factors were 0.192 (generalized linear model, *p* = 0.95, compared with 0.198 in Eq. 6) and 0.279 (*p* = 0.95, compared with 0.278 in Eq. 7). They are almost the same as the original analysis results, and the slope factor for KO is significantly larger than for WT mice as before (generalized linear model, *p* = 0.0003^a^). Equation 8 remained the same.

### Model of spine dynamics and the solution of the Langevin equation

The slow dynamics of *V* were modeled as the random variation defined using a Brownian motion (Eq. 9) within the range between the minimal (*V*_min_) and maximal (*V*_max_) values of 0.01 and 1.0 μm^3^, respectively, because spines with volume <0.01 or >1.0 μm^3^ were rare. Brownian motion (*W*) is the simplest random process, in which the mean is zero, but the variance is proportional to time. Using Brownian motion, a more general form of random processes with a mean *μ*(*V*) and variance *σ*(*V*), which depend on *V*, can be described by the following Langevin equation (Eqn. 9):

(9) $$\frac{dV(t)}{dt}=\sigma \left(V\left(t\right)\right)\frac{dW\left(t\right)}{dt}+\mu (V\left(t\right)),$$

where *W*(*t*) is the standard Brownian motion with a variance of 1 per 2 d. Following this, the transition probability density *p*(*V*, *t*|*y*) of the spine volume, which was *V* and *y* at time *t* and 0, respectively, satisfied the Fokker–Planck equation, if we performed stochastic integration according to Ito’s definition, as follows (Eqn. 10):

(10) $$\frac{\partial p(V,t|y)}{\partial t}=\frac{1}{2}\frac{\partial^{2}}{\partial V^{2}}\left[\sigma^{2}\left(V\right)p\left(V,t|y\right)\right]-\frac{\partial }{\partial V}\left[\mu \left(V\right)p\left(V,t|y\right)\right].$$

If a spine with the value of *V* bounces or disappears at the boundary, the boundary would be termed as reflecting or absorbing, respectively. How these boundaries are mathematically realized can be seen in the Mathematica (RRID:SCR_014448) notebook (Yasumatsu et al., 2008). The stationary solution of the Fokker–Planck equation can be derived directly, as follows, when a reflecting boundary condition is assumed at both *V*_min_ and *V*_max_ (Eqn. 11):

(11) $$f\left(V\right)=\frac{C}{\sigma^{2}(V)}Exp\left[\int \frac{2\mu (V)}{\sigma^{2}(V)}dV\right],$$

where *C* is a constant for normalization (Risken, 1984). The values of σ(*V*) and μ(*V*) were obtained from the experiments (Eqs. 6–8). Calculated *f*(*V*) was plotted to obtain the stationary distribution function of the spine volume, which is shown in Figure 7A.

The elimination rate of spines in *T* days was calculated as follows (Eqn. 12):

(12) $$E\left(T,f\right)=1-\int_{V_{min}}^{V_{max}}\int_{V_{min}}^{V_{max}}p\left(V,T|y\right)f\left(y\right)dydV,$$

where the stationary distribution *f* (*y*) as given above is assumed as the initial distribution and the absorbing boundary is set at *V* = *V*_min_.

### Statistical analyses

**Figure 6.**
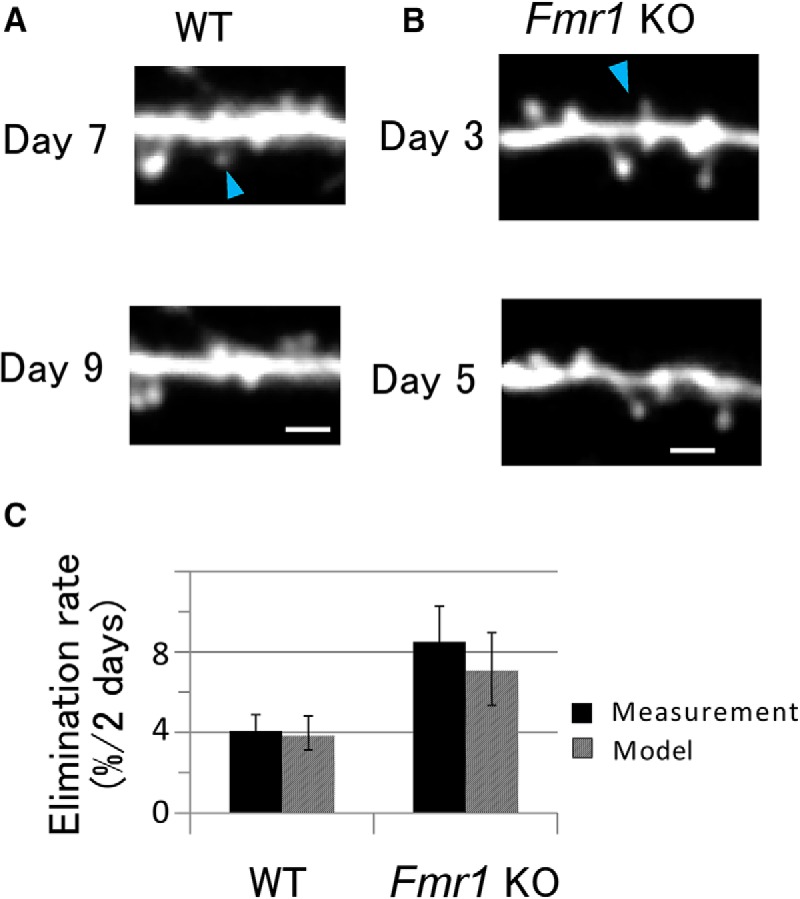
Elimination of dendritic spines per 2 d. ***A***, ***B***, Example of a time-lapse series of images of a dendrite in WT (***A***) and KO (***B***) mice at an interval of 2 d. Arrowheads indicate eliminated spines. Scale bar, 2 μm. ***C***, The elimination rates of dendritic spines per 2 d. Solid bars represent measurement data. Error bars represent SEM values from 6 and 12 dendrites for WT and *Fmr1* KO mice, respectively. KO mice seemed to have a higher elimination rate, although the difference was not significant (Mann–Whitney *U* test, *U* = 17, *p* = 0.08^b^). Hatched bars represent elimination rates calculated from the model shown with the dashed lines (Fig. 3*E*,*F*; Eqs. 6–8). Error bars represent 95% confidence intervals.

**Figure 7.**
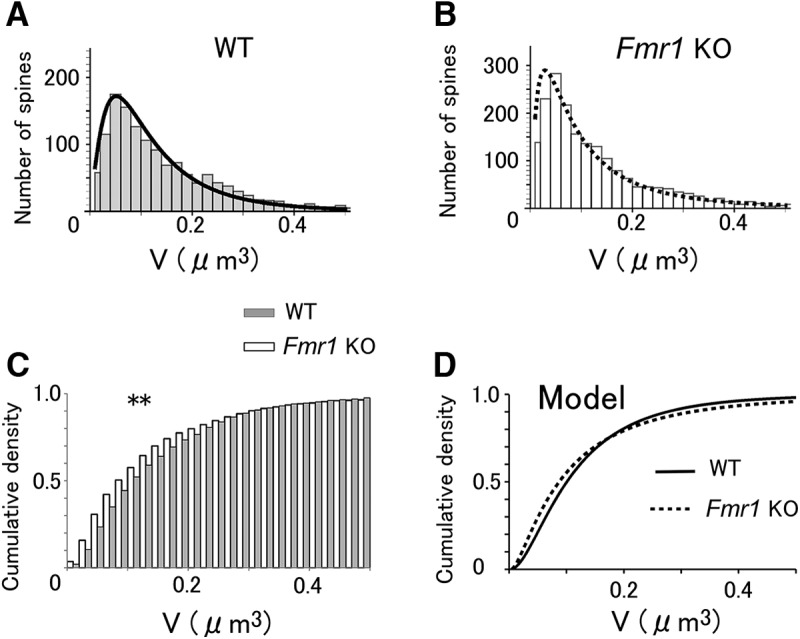
The volume distributions of spine-head volumes. ***A***, ***B***, The spine-head volume of WT (***A***) and KO (***B***) mice. The histogram represents data for 1368 spines (***A***) and 1913 spines (***B***). The lines represent a model fit by Equations 6, 7, and 11 based on dynamic data obtained (shown in Fig. 3*E*,*F*). ***C***, ***D***, Overlays of cumulative probability density distributions for WT and KO data (***C***) and models (***D***). The volume distribution of KO mice data is significantly different from that of WT mice. ** *p* = 0.003^c^ with the Kolmogorov-Smirnov test (χ^2^ = 13.1, df = 2).

A generalized linear model was used to compare the slopes of the fitted line in the plots of σ against *V* ^2/3^ (Fig. 3*E*,*F*). Spine elimination rates were compared using a Mann–Whitney *U* test (see Fig. 6*C*). The comparison of spine volume distribution was performed using a Kolmogorov–Smirnov test (see Fig. 7*C*; Table 1).

**Table 1:** Statistics

	Data structure	Type of test	Power
a	Not normal	Generalized linear model	Not applicable
b	Not normal	Mann–Whitney *U* test	Not applicable
c	Not normal	Kolmogorov–Smirnov test	Not applicable

### Code accessibility

The Mathematica notebook file of this study is available at https://doi.org/10.1523/ENEURO.0282-18.2018.f1-1. All code is also available as Extended Data 1.

10.1523/ENEURO.0282-18.2018.ed1 Extended Data 1 Code used for simulation of large-scale brain dynamics and scripts for subsequent postprocessing analyses. Download Extended Data 1, ZIP file.

## Results

### Dynamics of spine-head volumes

*In vivo* time-lapse imaging of the dendritic spines of the adult mouse visual cortex using two-photon microscopy was performed over 1 week to observe the longitudinal behavior of dendritic spines. A small glass window was made in the skull above the visual cortex of adult *Thy1*-GFP M mice, in which GFP was sparsely expressed, but was mainly found in layer V pyramidal neurons. A resonant scanner made it possible to obtain clearer images than those obtained with a conventional Galvano scanner in two-photon imaging (Fig. 1*A*). In fact, even in samples with considerable motion artifacts owing to heartbeats and respiration, the images were clear enough for spine volume measurements (Fig. 1*A*).

The analysis was restricted to layer I apical dendrites of layer V pyramidal neurons. Filopodia were excluded from the analysis (Materials and Methods), because they are thought to lack synaptic contacts and are expected to behave differently. Dendrites were imaged repeatedly at an interval of 2 d in WT and KO mice (Fig. 1*B*,*C*). Magnified images of two spines in Figure 1*C* are displayed in Figure 1*D*, showing that their heads are well defined. Spine volume was calculated from the images as explained in the Materials and Methods (Fig. 2*A*,*B*). Small spines were more likely to become larger (Fig. 2*C*,*D*, representing 754 spines from 15 dendrites in five WT mice and 878 spines from 20 dendrites in five KO mice), and large spines showed larger fluctuations in size, as observed in previous studies (Yasumatsu et al., 2008; Minerbi et al., 2009).

### Quantitative analysis of spine-head volume dynamics

Data from spines with similar volumes were pooled as explained in the Materials and Methods, and then the SD (Eq. 2) and the mean (Eq. 3) of the absolute volume changes per 2 d were calculated for quantitative evaluations. The dynamics expressed as the SD (σ) increased monotonically with increasing values of *V*, but the increase was sublinearly dependent on *V* (Fig. 3*A*), unlike what is observed in the young hippocampus (Yasumatsu et al., 2008). If we simply fit a line to σ (Fig. 3*A*,*B*, red lines), the linear slope factors were 0.21 and 0.32 for WT and *Fmr1* KO mice, respectively. The linear fits, however, showed relatively large errors for small spines (<0.06 μm^3^), indicating that the small spines fluctuated less than was linearly predicted using *V* for all spines.

A double logarithmic plot showed that the sublinearity was *V* ^0.6^ (Fig. 3*C*,*D*). Therefore, a value of two-thirds was chosen to represent this sublinearity, to allow an alternative interpretation of *V* ^2/3^, instead of the volume *V*, as the surface area of the spine. Subsequently, σ was plotted against *V* ^2/3^ (Fig. 3*E*,*F*, filled circles). Since the behavior of very small spines has an important influence on the spine turnover, we anchored the line to a point at *V* = 0.015 (the median value of the smallest bin) and σ = 0.02 (average value of σ for the smallest bin), and obtained a suitable slope for the straight line (σ vs *V* ^2/3^) from the observed data, as shown with the solid lines. The values of σ remained positive near *V* = 0, corresponding to the observations that even very small spines showed a certain amount of dynamics, as seen in the scatter chart (Fig. 2*C*,*D*). The difference in the slope between WT and KO mice is statistically significant (generalized linear model, *p* = 0.0006^a^), indicating that the volume dynamics of spines are significantly greater in KO than in WT mice.

Similarly, the mean of the spine volume that changed per 2 d (μ, drift) was plotted against *V* ^2/3^ (Fig. 3*G*,*H*). The values of μ decreased monotonously with an increase in the spine volume and showed a value of zero at *V* = 0.12. Spines smaller than this volume tended to increase in size, as per the scatter chart. Since the means of spine volume changes were similar between the WT and KO mice (Fig. 3, compare *G*, *H*), they were fitted with the same equation (Equation 8).

### Fast and slow dynamics

The dendritic spines displayed fast fluctuations, which occurred independently of the slow dynamics that determined spine elimination and volume distributions. To estimate the fast dynamics in the adult mice, we performed time-lapse imaging with an interval of 10 min for 1 h (Fig. 4*A*,*B*). These observations cannot simply reflect measurement errors (Mysore et al., 2007; Yasumatsu et al., 2008), because such fluctuations were not observed in the fixed brain preparations even after a 2 d interval (Fig. 4*C*; generalized linear model, *p* = 0.00002^a^). Since the fast dynamics (Fig. 4*D*,*E*) were similar between the WT and KO mice (Fig. 5, compare *A*, *B*), they were fitted with the same equation (Equation 4). The means of the spine volume changes were negligible for both the WT and KO mice (Fig. 5*C*,*D*), and were represented by zero. Since these fluctuations mainly reflect actin-dependent rapid fluctuations (Honkura et al., 2008), we simply expressed these using variances and means (Fig. 5*A–D*). Using the fast dynamics and Equation 5, the slow dynamics were obtained with Equations 6 and 7, as shown in the dashed lines in Figure 3, *E* and *F*.

### Spine turnovers

Spine elimination was measured using time-lapse imaging with an interval of 2 d, and was expressed as the percentage of the spines that disappeared in the observation period. Figure 6, *A* (WT) and *B* (KO), shows examples of the spine eliminations occurring in the 2 d interval. The elimination rates observed were 4.1% (WT) and 8.5% (KO) per 2 d (Fig. 6*C*; Mann–Whitney *U* test, *p* = 0.08^b^), which is consistent with a previous observation (Nagaoka et al., 2016) that the elimination rate was significantly higher in KO mice (Steel’s test, *p* < 0.01).

Predictions for spine elimination rates using the model were performed by replacing the boundary condition at the minimum spine volume from a reflecting boundary to an absorbing boundary, as explained in the Materials and Methods. The predicted rates could then be compared with the measured elimination rates. Brownian motion describes the behavior of a time-dependent random variable, *V*(*t*), with an SD, σ(*V*), and a drift, μ(*V*), as the stochastic process, as per Equations 9 and 10. Using Equation 12, the elimination rate for WT mice was calculated to be 3.8%, which is consistent with the measured rate of 4.1% (Fig. 6*C*). Similarly, for the KO mice, the predicted rate was 7.1%, which is consistent with the measured rate of 8.5%.

### Statistical distribution of spine volume

Experimentally observed volume distributions in WT and KO mice were significantly different (Kolmogorov–Smirnov test, *p* = 0.003^c^; Fig. 7*A–C*). Interestingly, small spines (<0.06 µm^3^) in KO mice were more prevailing than those in WT mice, while the medium-sized spines were less abundant, and the very large spines (e.g., >0.3 µm^3^) tended to be more abundant in KO mice (Fig. 7*C*), although the difference was not statistically significant. Consequently, the average volume for the WT mice was 0.152 ± 0.003 µm^3^ (mean ± SEM), and for the KO mice it was 0.144 ± 0.003 µm^3^, and these values were not significantly different (*p* = 0.09, Student’s *t* test).

We predicted the spine volume distribution from the volume dynamics of spines, as was done in a previous study (Yasumatsu et al., 2008). The values of σ and μ were derived from the experimental data as the SD (Eq. 2) and the mean (Eq. 3) of the slow dynamics of the spine volume (Fig. 3*E*,*F*). The stationary distributions function *f*(*V*) of the spine volume could be calculated with Equation 11 (Risken, 1984), with a reflecting boundary condition at both the minimum (*V*_min_ = 0.01 µm^3^; corresponding to the smallest spines observed) and maximum (*V*_max_ = 1.0 µm^3^; only a few spines are larger than this) spine volumes. Figure 7, *A* (WT) and *B* (KO), show that the calculated *f*(*V*) (smooth lines) fits with the experimentally observed spine volume histograms (1368 spines for WT, 1913 spines for KO). In particular, the model accounts for the abundance of small and large spines in KO mice (Fig. 7*D*), but not for the medium-sized spines, which is in line with the experimental observations. The model estimated the average spine volumes to be 0.134 and 0.137 µm^3^ for WT and KO mice, respectively; these values were similar to the experimental observations.

## Discussion

### Dynamics of spine volumes in the neocortex of WT and *Fmr1* KO mice

We performed *in vivo* imaging of dendritic spines in the adult mouse visual cortex using a resonant scan two-photon confocal microscope. The elimination rate of spines in the young hippocampus was 8%/d (Yasumatsu et al., 2008), but it was low in the neocortex (8%/14 d; Zuo et al., 2005). Assuming the same linear model for σ, the fluctuations must be attenuated in the neocortex to 0.27 (=1/$\sqrt{14}$) times the slope factor of that in young hippocampal preparations (Yasumatsu et al., 2008). We found, however, that when the values of σ were fitted with linear functions of *V*, the slope factor was attenuated only to 0.75 times [=0.21/0.28, where 0.21 is the slope of the straight red line in Figure 3*A*; 0.28 is the corresponding slope for the hippocampus from the previous report (Yasumatsu et al., 2008)]. This implies that the mathematical model developed for the hippocampus cannot be simply generalized to the neocortex. Instead, we found that the SD σ of the spine volume dynamics was proportional to *V* ^2/3^, and not *V*, in the neocortex *in vivo* (Fig. 3*E*,*F*; Eqs. 6 and 7). Owing to the sublinearity, the actual values of σ for small spines were smaller than those predicted from the linear fitting (Fig. 3*A*,*B*, red lines). Thus, the small spines in the neocortex are less dynamic than can be predicted from the linear (or multiplicative) dependence of the dynamics of spine volumes.

It is unclear why the values of σ were proportional to the surface area (*V* ^2/3^), while σ depended linearly on *V* in the young hippocampus. One reason for the variation in spine stability is that this factor may depend more on the extracellular matrix (ECM) in the adult mouse neocortex, and the surface structures of spines that contact the ECM play a more important role than do the cytosolic volume factors. Such stabilizing mechanisms are also active in *Fmr1* KO mice, as the dynamics of their spines also fit our sublinear model. The slope factor of 0.28 in *Fmr1* KO mice, compared with the slope factor of 0.20 in WT mice, could quantitatively account for the increases in the elimination rates of the spines. The unusually high expression of an ECM proteinase, matrix metalloproteinase-9, in *Fmr1* KO mice may contribute to the greater slope factor observed in these mice (Sidhu et al., 2014; Reinhard et al., 2015; Nagaoka et al., 2016).

### Spine volume-related parameters in the neocortex of WT and *Fmr1* KO mice

Our *in vivo* study revealed that spine volumes showed apparently random fluctuations, akin to what would be observed if there were errors in the measurements, but the model developed using the volume dynamics well explained the observed values of volume distributions and spine turnovers in both WT and *Fmr1* KO mice. This indicates that the observed dynamics of the spine volumes are the basis of the observed spine volume distributions. Interestingly, the spine volume distributions in *Fmr1* KO and WT mice showed a complex dependence on the spine volume: small spines were more frequently observed in *Fmr1* KO mice, but the medium-sized spines were less abundant. Moreover, large spines may be more abundant in *Fmr1* KO mice than in their WT counterparts (Fig. 7*C*). This is the mathematical consequence of the larger slope factor in *Fmr1* KO mice (Fig. 7*D*), which tends to sharpen the peak of the spine volume and then increase the tail of the distribution as a compensatory measure. Due to this secondary factor, the average spine volumes were similar (WT mice, 0.152 µm^3^; KO mice, 0.144 µm^3^). The subtle difference in the averaged volume could be one reason why spine volume changes are often overlooked (Nimchinsky et al., 2001; Wijetunge et al., 2014; but see Suresh and Dunaevsky, 2017).

The complex nature of the effects of *Fmr1* KO on spine volume distribution can be revealed only with a large sample size and adequate estimation of spine volumes using spine intensity in this and a previous study (Suresh and Dunaevsky, 2017), instead of spine width (Wijetunge et al., 2014). Despite the small difference in the average spine volume, the spine dynamics are severely impaired in *Fmr1* KO mice even in adulthood, as is reflected in the increase in turnover rates (Pan et al., 2010; Padmashri et al., 2013; Nagaoka et al., 2016) and abnormal behaviors (Consortium-D-BFX, 1994; Padmashri et al., 2013).

In the present study, we did not distinguish between activity-dependent and activity-independent intrinsic dynamics (Yasumatsu et al., 2008; Minerbi et al., 2009; Kasai et al., 2010; Loewenstein et al., 2015; Nagaoka et al., 2016; Chambers and Rumpel, 2017; Mongillo et al., 2017; Okazaki et al., 2018; Ziv and Brenner, 2018). Since the spine elimination rates are not significantly affected by the blockage of NMDA receptors and voltage-dependent calcium channels under our conditions both in WT and *Fmr1* KO mice (Nagaoka et al., 2016), it is possible that the slow dynamics measured here reflect activity-independent intrinsic dynamics to a large extent, if not completely. In the future, it will be interesting to test whether the increase in the slope factor is primarily due to the intrinsic dynamics, and to elucidate how the slope factor is determined.

## Synthesis

Reviewing Editor: Pablo Castillo, Albert Einstein College of Medicine Decisions are customarily a result of the Reviewing Editor and the peer reviewers coming together and discussing their recommendations until a consensus is reached. When revisions are invited, a fact-based synthesis statement explaining their decision and outlining what is needed to prepare a revision will be listed below.

The reviewers understand the authors have properly addressed most of their concerns but the revised manuscript falls short in addressing the impact of a fresh craniotomy on the spine volume dynamics. As discussed online and clearly indicated in the reviewers' specific comments (see below), it is essential that you directly address this issue not only by explaining how the surgery was performed but also by reanalyzing the existing data while excluding data from day one post-surgery. It is hard to believe there is no tissue damage right after surgery.

REVIEWER #1

The manuscript has been greatly improved by a thorough description of experimental methods and the clarification of data interpretation. The authors have addressed all my original concerns. Some minor comments:

1. Figs. 1 and 2 are closely related to each other, and it could be good to combine them to one figure.

2. Fig. 3 E-H, please check the label for X axis. Is it V or V2/3?

3. Lines 270-273; Fig. 4A-C. Although data in Fig. 4A-B appear different compared to Fig. 4C, the authors need to present statistical analysis to support their statement that spine volume fluctuations over 10 min (the fast dynamics) are not simply due to measurement errors.

REVIEWER #2

The authors are making a point that there is a relationship between spine volume fluctuations and spine turnover rates that could explain the higher turnover rate of spines in the KO mouse. However, the authors explain that they are not determining this relationship on the level of individual spines because of the small number of spines as well as imaging parameters (laser power). This major conclusion of the study is therefore no fully supported.

Although, it is appreciated that the surgery is made by an expert, it is well known that a cranial window is an invasive approach. Providing more details of the surgical procedure does not alleviate this concern. Since the authors have the data in hand it is not clear why comparisons of spine dynamics cannot be made between the first few days after surgery and that observed 2-3 weeks after.

The authors explain in the rebuttal that the description of imaging period of "one week" in the abstract is representative of most of the data. This is not going to be clear to a reader of this manuscript and is not congruent with what is described in the text. This is somewhat messy.

For figure 1, the inset showing that the spines are actually not stubby is not very convincing.

## Author Response

Synthesis of Reviews:

Significance Statement Comments for Author (Required):

n/a

Synthesis Statement for Author (Required):

The reviewers understand the authors have properly addressed most of their concerns but the revised manuscript falls short in addressing the impact of a fresh craniotomy on the spine volume dynamics. As discussed online and clearly indicated in the reviewers' specific comments (see below), it is essential that you directly address this issue not only by explaining how the surgery was performed but also by reanalyzing the existing data while excluding data from day one post-surgery. It is hard to believe there is no tissue damage right after surgery.

Thank you so much for the synthesis statement. We performed the reanalysis as suggested. The results are briefly stated in the paper. The slope factors were 0.192 (generalized linear model, p=0.95, compared to 0.198 in equation 6) in WT and 0.279 (p=0.95, compared to 0.278 in equation 7) in KO mice, and the slope factor for KO is significantly larger than for WT mice as before (generalized linear model, p=0.0003a). This indicates that the central thesis of the paper (σWT&lt;σKO) remains valid. This is now stated in the paper (lines 180-185).

-----------------------------------------------------------------------

REVIEWER #1

The manuscript has been greatly improved by a thorough description of experimental methods and the clarification of data interpretation. The authors have addressed all my original concerns. Some minor comments:

1. Figs. 1 and 2 are closely related to each other, and it could be good to combine them to one figure.

Thank you for valuable comments again. Since we have now added one more panel to the Fig. 1 in response to a comment of the reviewer #2, and since Figs. 2A-B and Figs. 2C-D should be shown together, there is not enough space to combine Fig. 1 and 2. We have thus decided to show the Figs. 1B-C and Figs. 2A-B separately.

2. Fig. 3 E-H, please check the label for X axis. Is it V or V2/3?

We understand the concern. The X-axis actually reflect V2/3, but the numbers and scales along the axis represent V instead. We decided to do so, because displaying the numbers by V2/3 is confusing. The figure legends are altered to make this point clear (lines 532 and 541-2).

3. Lines 270-273; Fig. 4A-C. Although data in Fig. 4A-B appear different compared to Fig. 4C, the authors need to present statistical analysis to support their statement that spine volume fluctuations over 10 min (the fast dynamics) are not simply due to measurement errors.

A statistical analysis using generalized linear model was performed to confirm the difference. It is added in the Results section (line 282).

REVIEWER #2

The authors are making a point that there is a relationship between spine volume fluctuations and spine turnover rates that could explain the higher turnover rate of spines in the KO mouse. However, the authors explain that they are not determining this relationship on the level of individual spines because of the small number of spines as well as imaging parameters (laser power). This major conclusion of the study is therefore no fully supported.

Thank you so much for careful reading of our manuscript again.

We apologize for having made confusing statements. The spines used for the measurement of turnover rate are indeed subsets of the spines used for the measurement of volume fluctuations. This was inevitable because the laser power had to be kept low to avoid saturation. We think this does not pose a problem because the subsets are nearly a random sample of the whole data set, selected based on the absence of the very bright dendritic spines or shafts. In addition, the turnover rates were similarly estimated as in the previous study (Nagaoka et al., 2016). We have added a sentence to clarify the selection process (lines 146-147): “Images not bright and clear enough to enable unambiguous distinction were excluded from the measurement of elimination rates.”

Although, it is appreciated that the surgery is made by an expert, it is well known that a cranial window is an invasive approach. Providing more details of the surgical procedure does not alleviate this concern. Since the authors have the data in hand it is not clear why comparisons of spine dynamics cannot be made between the first few days after surgery and that observed 2-3 weeks after.

We performed the reanalysis as suggested in the synthesis statement. The results show that the central thesis of the paper (σWT&lt;σKO) remains valid. This is now stated in the paper (lines 180-185): “In order to reassure that the operation itself did not affect the results, the analysis was performed excluding the imaging data from day 1 post-surgery. The slope factors were 0.192 (generalized linear model, p=0.95, compared to 0.198 in equation 6) and 0.279 (p=0.95, compared to 0.278 in equation 7). They are almost the same as the original analysis results, and the slope factor for KO is significantly larger than for WT mice as before (generalized linear model, p=0.0003a). The Equation 8 remained the same.”

The authors explain in the rebuttal that the description of imaging period of “one week” in the abstract is representative of most of the data. This is not going to be clear to a reader of this manuscript and is not congruent with what is described in the text. This is somewhat messy.

The range of the imaging period is now stated in line 93-94, and omitted from the Abstract to avoid confusion. Thank you for pointing out this potential issue.

For figure 1, the inset showing that the spines are actually not stubby is not very convincing.

The inset is now magnified and shown in a separate panel (Fig. 1D) for a clearer view. Since the spine heads can be readily discerned and the ROIs can be placed, they were included in the volume measurement.
